# Growth Transformation of B Cells by Epstein-Barr Virus Requires *IMPDH2* Induction and Nucleolar Hypertrophy

**DOI:** 10.1128/spectrum.00440-23

**Published:** 2023-07-06

**Authors:** Atsuko Sugimoto, Takahiro Watanabe, Kazuhiro Matsuoka, Yusuke Okuno, Yusuke Yanagi, Yohei Narita, Seiyo Mabuchi, Hiroyuki Nobusue, Eiji Sugihara, Masaya Hirayama, Tomihiko Ide, Takanori Onouchi, Yoshitaka Sato, Teru Kanda, Hideyuki Saya, Yasumasa Iwatani, Hiroshi Kimura, Takayuki Murata

**Affiliations:** a Department of Virology, Fujita Health University School of Medicine, Toyoake, Japan; b Clinical Research Center, National Hospital Organization Nagoya Medical Center, Nagoya, Japan; c Department of Virology, Nagoya University Graduate School of Medicine, Nagoya, Japan; d Department of Virology, Nagoya City University Graduate School of Medical Sciences, Nagoya, Japan; e Division of Infectious Disease, Department of Medicine, Brigham and Women's Hospital and Harvard Medical School, Boston, Massachusetts, USA; f Department of Pathology and Laboratory Medicine, Nagoya University Hospital, Nagoya, Japan; g Division of Gene Regulation, Cancer Center, Research Promotion Headquarters, Fujita Health University, Toyoake, Japan; h Department of Morphology and Diagnostic Pathology, School of Medical Sciences, Fujita Health University, Toyoake, Japan; i Department of Biomedical Molecular Sciences, Graduate School of Medicine, Fujita Health University, Toyoake, Japan; j Open Facility Center, Research Promotion Headquarters, Fujita Health University, Toyoake, Japan; k Department of Microbiology, Faculty of Medicine, Tohoku Medical and Pharmaceutical University, Sendai, Japan; Barnard College, Columbia University

**Keywords:** EBV, IMPDH2, nucleolar hypertrophy, growth transformation, MPA, MMF

## Abstract

The *in vitro* growth transformation of primary B cells by Epstein-Barr virus (EBV) is the initial step in the development of posttransplant lymphoproliferative disorder (PTLD). We performed electron microscopic analysis and immunostaining of primary B cells infected with wild-type EBV. Interestingly, the nucleolar size was increased by two days after infection. A recent study found that nucleolar hypertrophy, which is caused by the induction of the *IMPDH2* gene, is required for the efficient promotion of growth in cancers. In the present study, RNA-seq revealed that the *IMPDH2* gene was significantly induced by EBV and that its level peaked at day 2. Even without EBV infection, the activation of primary B cells by the CD40 ligand and interleukin-4 increased *IMPDH2* expression and nucleolar hypertrophy. Using *EBNA2* or *LMP1* knockout viruses, we found that *EBNA2* and *MYC*, but not *LMP1*, induced the *IMPDH2* gene during primary infections. IMPDH2 inhibition by mycophenolic acid (MPA) blocked the growth transformation of primary B cells by EBV, leading to smaller nucleoli, nuclei, and cells. Mycophenolate mofetil (MMF), which is a prodrug of MPA that is approved for use as an immunosuppressant, was tested in a mouse xenograft model. Oral MMF significantly improved the survival of mice and reduced splenomegaly. Taken together, these results indicate that EBV induces *IMPDH2* expression through *EBNA2*-dependent and *MYC*-dependent mechanisms, leading to the hypertrophy of the nucleoli, nuclei, and cells as well as efficient cell proliferation. Our results provide basic evidence that *IMPDH2* induction and nucleolar enlargement are crucial for B cell transformation by EBV. In addition, the use of MMF suppresses PTLD.

**IMPORTANCE** EBV infections cause nucleolar enlargement via the induction of IMPDH2, which are essential for B cell growth transformation by EBV. Although the significance of IMPDH2 induction and nuclear hypertrophy in the tumorigenesis of glioblastoma has been reported, EBV infection brings about the change quickly by using its transcriptional cofactor, EBNA2, and MYC. Moreover, we present here, for the novel, basic evidence that an IMPDH2 inhibitor, namely, MPA or MMF, can be used for EBV-positive posttransplant lymphoproliferative disorder (PTLD).

## INTRODUCTION

Epstein-Barr virus (EBV) is a gamma-herpesvirus that is also known as the human tumor virus. It is associated with various cancers, such as Burkitt lymphoma, Hodgkin lymphoma, NK/T cell lymphoma, chronic active EBV infection, nasopharyngeal carcinoma, and gastric carcinoma (1, 2). It possesses a linear double-stranded DNA genome of approximately 175 kbps. The virus is transmitted via saliva, typically during childhood, and it is present in >90% of adults. EBV infection may cause infectious mononucleosis.

Its infections may be latent and lytic. The conversion from the former to the latter is called reactivation (3). In lytic infections, EBV expresses its genes in a well-coordinated manner, amplifies its viral genome in the nuclear replication compartments, and produces progeny virions. In latent infections, the EBV genome is synthesized in the S phase by using host replication machinery without the production of viral particles; in this stage, EBV expresses only a small subset of viral genes, namely, six EBV nuclear antigens (EBNA1, 2, 3A, 3B, 3C, and LP), three latent membrane proteins (LMP1, 2A, and 2B), two EBV-encoded small RNAs (EBERs), and 44 viral microRNAs (4).

EBV efficiently infects primary human B lymphocytes and induces cellular growth transformation, thereby resulting in immortalized cells that proliferate continuously. This is called the lymphoblastoid cell line (LCL). This reprogramming of resting B cells has been studied extensively and represents, at least partly, lymphomatogenesis by EBV. *EBNA2*, *3A*, *3C*, and *LMP1* genes are essential, and *EBNA1*, *LP*, and *LMP2A* are also important for B cell transformation and the continuous proliferation of infected B cells by EBV (5). Of these genes, the *EBNA2* gene is the most important because *EBNA2*-knockout EBV does not initiate transformation (6). The *EBNA2* gene encodes a powerful transcriptional cofactor that acts as a complex with RBPJ and certain other transcription factors, thereby activating the transcription of key viral and cellular genes immediately after an infection. Recent transcriptome analyses have demonstrated that EBNA genes, including *EBNA2*, are expressed immediately and are maintained at high levels after an EBV infection of primary B cells. In contrast, the expression of the LMP genes, such as *LMP1*, is limited for a few days after the infection, and this is followed by a gradual increase for weeks and then by the maintenance of levels (7). Viral lytic genes are also expressed after an EBV infection without viral DNA synthesis, and they are silenced after weeks or even months. Lytic gene expression before latent infection is termed the pre-latent abortive lytic phase or the pre-latent phase (8, 9). Primary B cells that are infected with EBV efficiently proliferate as LCL, expressing EBNAs and LMPs, and have limited expression of lytic genes. This pattern is called latency III. In addition to viral genes, the expression of cellular genes is also widely and severely affected (10–12). For instance, the prompt induction and sustained high expression of *MYC* is essential for B cell growth transformation by EBV. The *MYC* gene is induced in a super-enhancer-dependent manner that involves *EBNA2* and other cellular transcription factors, such as NF-κB (13).

The B cell transformation process has been investigated based on morphological changes. Microscopy and fluorescence-activated cell sorting analyses of primary B cells has demonstrated that nuclei and cells become markedly bigger after an EBV infection (10, 14, 15). The mutagenesis of the EBV genome has shown that *EBNA2* plays an essential role in this size change and in the early activation of B cells; *EBNA-LP*, *LMP2A*, and viral microRNAs play supportive roles in the process, whereas *LMP1*, *EBNA3A*, *3C*, and *EBERs* have minimal effects (14). Because LCLs are associated with enlarged nuclei and cells, it is hypothesized that this increase may be required for B cell transformation by EBV. However, the exact molecular mechanism and the significance of the enlargement of cells and nuclei by *EBNA2* during transformation is unclear.

In the present study, we performed transmission electron microscopy (TEM) and confocal microscopy, combined with three-dimensional (3D) reconstruction, and found that the nucleolar size and number were increased in primary B cells within a few days after an EBV infection. We also found that the expression of inosine-5′-monophosphate dehydrogenase 2 (*IMPDH2*), which is the rate-limiting enzyme for *de novo* GTP synthesis, was induced by day 2. This induction was required for nucleolar growth and the increased sizes of the nuclei and cells. *IMPDH2* induction depended on *EBNA2* and *MYC*. Pharmaceutical *IMPDH2* inhibition prevented EBV-driven growth transformation and resulted in prolonged survival in a mouse xenograft model. Notably, the stimulation of B cell proliferation by the CD40 ligand (CD40L) and interleukin-4 (IL-4) was associated with *IMPDH2* induction and nucleolar hypertrophy. Therefore, *IMPDH2* is a key molecule that is involved in nucleolar hypertrophy in B cell growth transformation by EBV and also in proliferation in the absence of EBV. These results suggest that *IMPDH2* inhibitors may be used as immunosuppressants in transplant patients to prevent posttransplant lymphoproliferative disorder (PTLD).

## RESULTS

### Morphological changes in B cells after EBV infection.

**FIG 1:**
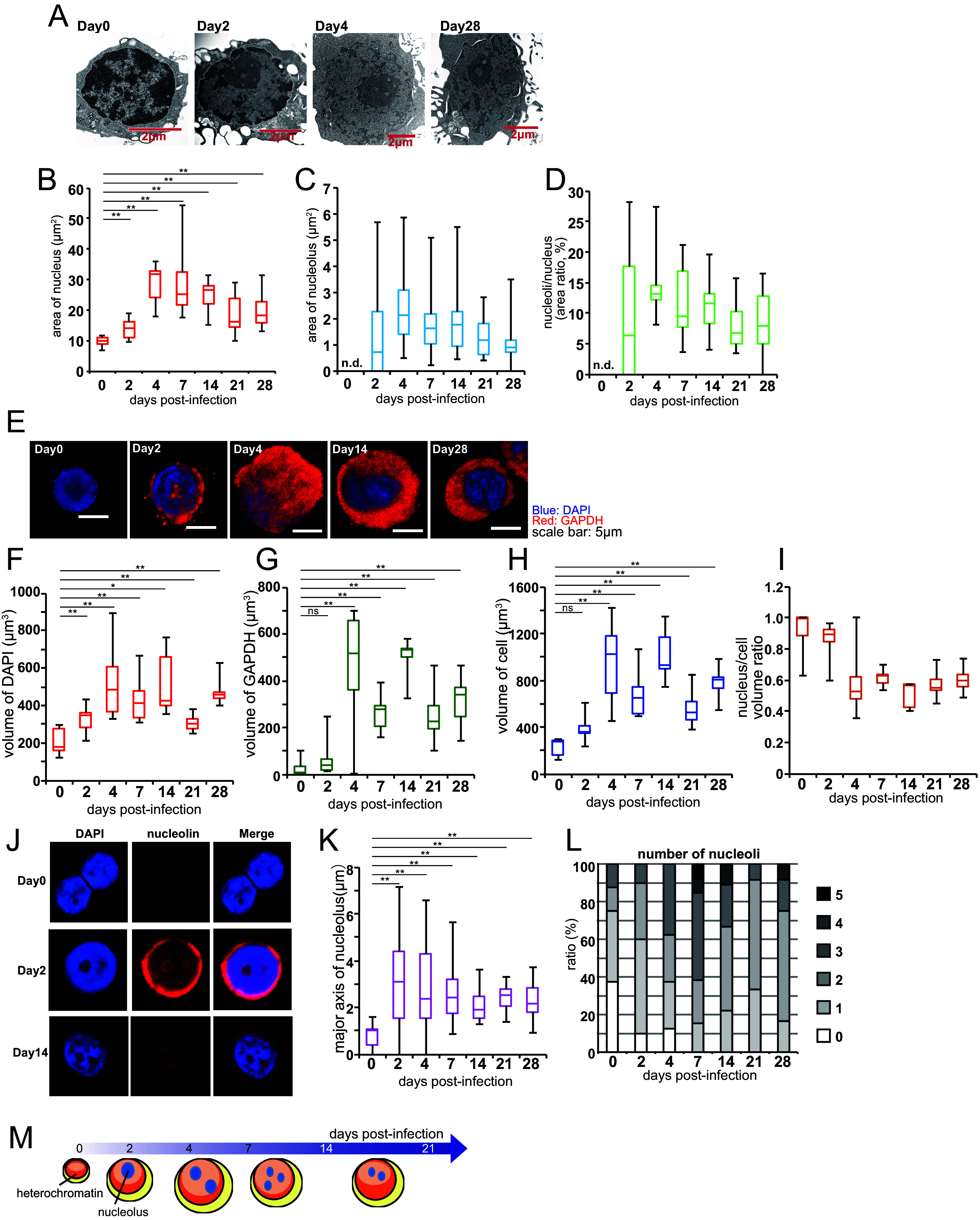
Nuclear, cellular, and nucleolar hypertrophy caused by the EBV infection of primary B cells. (A–L) Primary B cells were infected with EBV at a MOI of 3 and harvested at the indicated time points. (A–D) Typical images of EBV-infected cells were analyzed via TEM (A), and morphological changes are shown in box and whisker plots (B–D). Areas of nucleus (B) and nucleolus (C) were quantified using the ImageJ software package (*n* = 10 to 15). The nucleoli/nucleus area ratio is shown in panel D. The n.d. abbreviation indicates not detected. (E–L) Morphology was analyzed using immunofluorescence assays. Serial stacked sections were photographed using an LSM710 confocal laser scanning microscope. The images were reconstituted for three-dimensional visualization using the Imaris software package for the quantification of volumes (*n* = 10 to 15). (E and J) Representative images of EBV-infected cells. (F–I) The volumes of nucleus (panel F, DAPI), cytoplasm (panel G, GAPDH), and cell (panel H, DAPI + GAPDH) as well as the nucleus/cell volume ratio (I) are shown in box and whisker plots. (K) The major axes of nucleoli (fibrillarin) were measured using the Zen software package. (L) Number of nucleoli per cell. (M) Graphical summary of the morphological changes of the EBV-infected B cells. At 2 to 4 dpi, the nucleus and cytoplasm of the EBV-infected cells increased in size by almost twofold to fourfold, compared to noninfected cells (0 dpi). Nucleolar enlargement reached its maximum at 2 to 4 dpi. The number of nucleoli increased from 4 to 14 dpi and reached its maximum at 7 dpi. Two-tailed Student’s *t* tests were used to indicate between-group differences. Ns, not significant; *, *P* < 0.05; **, *P* < 0.01.

To analyze the morphological changes in EBV-infected B cells during growth transformation, primary B cells were infected with EBV at a multiplicity of infection (MOI) of 3. The cells were harvested at the indicated time points after infection for a TEM analysis. At day 0 postinfection (dpi), a large area of the peripheral nuclear region was enriched with dark heterochromatin, which is a typical feature of resting B cells, and the nucleolus was not evidently detectable (Fig. 1A). However, the heterochromatin structure receded after infection, likely representing hypomethylation (3, 16–18). The size of the nuclei and cells increased after EBV infection (Fig. 1A), as reported previously (11, 14). The nuclear size increased by twofold to fourfold at 4 to 14 dpi and then gradually decreased, although it remained larger than that observed at 0 dpi (Fig. 1B). The nucleolar size remained increased from that observed at 2 dpi (Fig. 1A). The nucleolar area was at its maximum (about 2 μm^2^ per nucleolus, on average) at 4 dpi, and this was followed by a slight decrease (Fig. 1C). However, the nucleolar areas remained enlarged. The proportion of nucleolar area in the nuclear area increased immediately after infection and peaked at 4 dpi (Fig. 1D).

We performed immunofluorescence assays using DAPI, anti-GAPDH, and anti-nucleolin or anti-fibrillarin antibodies to visualize the nucleus, cytoplasm, and nucleolus, respectively. The stained sections were observed using confocal microscopy and 3D reconstruction to quantify the nuclear/cellular volumes and nucleolar major axes (Fig. 1E–K). The nuclear volume peaked at 4 dpi, and this was followed by a slight decrease (Fig. 1F), although there was some variation in volume. The cytoplasm volume was hardly detectable at 0 dpi, but it peaked at 4 dpi and slightly decreased thereafter (Fig. 1G). The cell volume (nucleus plus cytoplasm) was at its highest at 4 dpi and slightly decreased thereafter (Fig. 1H), whereas the volume ratio of the nucleus to the cell did not increase and, in fact, decreased slightly (Fig. 1I), likely because of the greater increase in cell volume than in the nuclear volume. A similar experiment was performed with nucleolar staining (Fig. 1J) to measure the major axis of the nucleoli. The results (Fig. 1K) were similar to those obtained from the TEM (Fig. 1C).

In addition, we counted the number of nucleoli in the nuclei by using 3D images (Fig. 1L). Noninfected primary B cells typically possessed either no nucleoli or one nucleolus (day 0), but EBV infection led to an increase in the number of nucleoli, which reached its maximum at 7 dpi and was followed by a slight decrease to 2 to 4 nucleoli per cell at 28 dpi.

Our findings are summarized in Fig. 1M. The nucleolar size increased as early as 2 dpi and slightly decreased after 4 dpi (Fig. 1K). EBV infection led to more nucleoli per cell, reaching its maximum at 7 dpi (Fig. 1L). The transformed cells (at 28 dpi) had more and larger nucleoli than did the noninfected cells (0 dpi). The heterochromatin domain receded at 2 dpi and persisted thereafter (Fig. 1A). The nuclear and cellular sizes were the largest at 4 or 14 dpi (Fig. 1F–H), indicating that nucleolar enlargement occurred earlier than nuclear and cellular enlargement.

### Role of *IMPDH2* in nucleolar hypertrophy.

**FIG 2:**
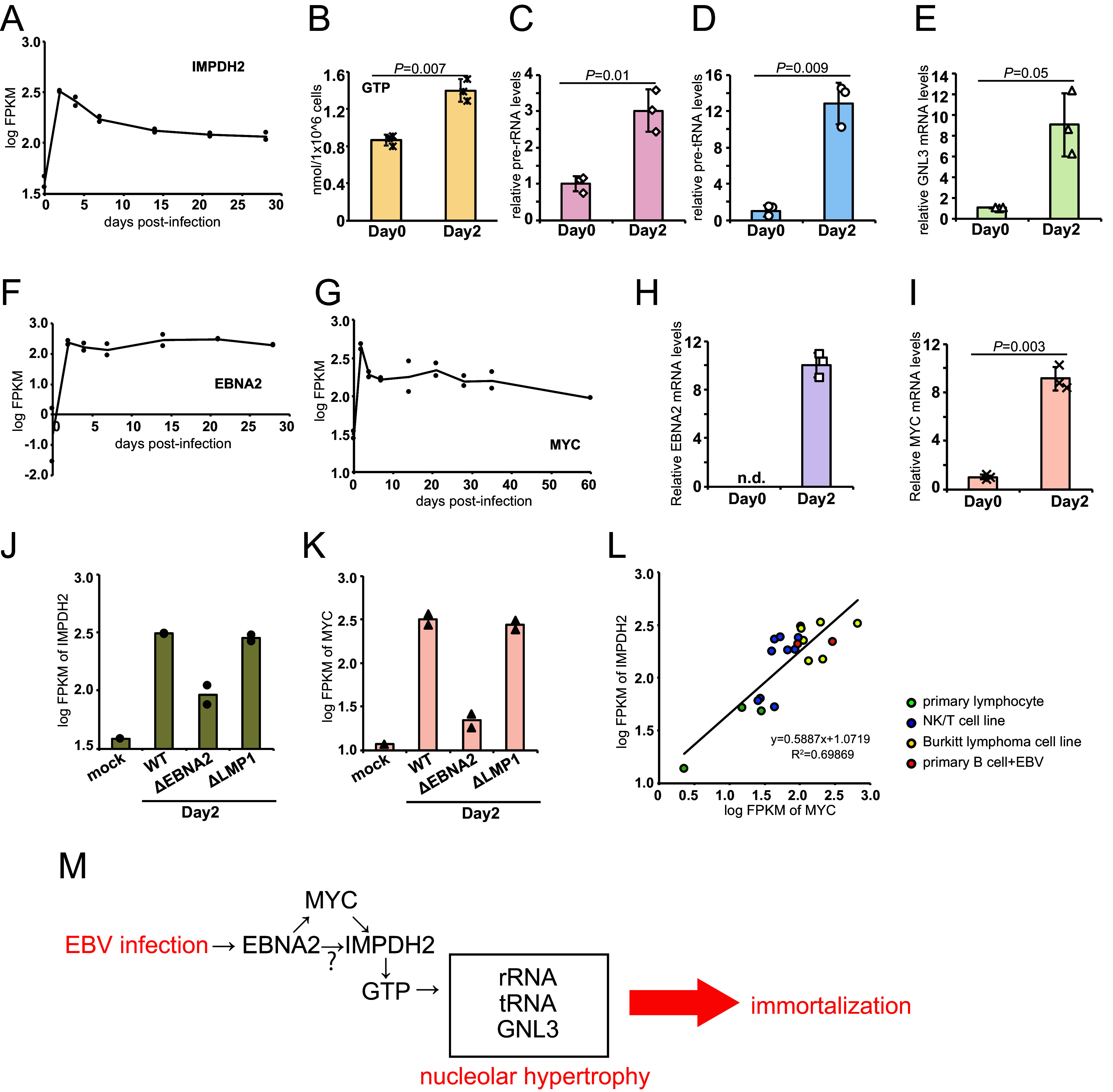
EBV infection of primary B cells caused the *EBNA2*-dependent induction of *IMPDH2* and increased GTP, pre-rRNA, and pre-tRNA levels. (A, F, and G) Induction of *IMPDH2* (panel A), *EBNA2* (panel F), and *MYC* (panel G) transcription by EBV infection. Primary B cells were infected with EBV at a MOI of 3, and RNA was harvested at the indicated time points for RNA-seq. (B) GTP levels in EBV-infected cells at 0 and 2 dpi. (C–E, H, and I) RT-qPCR analyses for pre-rRNA (panel C), pre-tRNA (panel D), *GNL3* (panel E), *EBNA2* (panel H), and *MYC* (panel I) in EBV-infected cells at 0 and 2 dpi. (J and K) *EBNA2*-dependent induction of *IMPDH2* (panel J) and *MYC* (panel K). Primary B cells were infected with EBV (wild-type, d*EBNA2*, or d*LMP1*), and RNA was harvested at 2 dpi for RNA-seq. (L) Correlation between the expressions of *IMPDH2* and *MYC*. RNA was purified from each cell line or from primary B cells for RNA-seq. The logFPKM values are presented in Table 1. Correlations were examined via a Pearson correlation analysis. (M) Graphical summary of *IMPDH2* induction and immortalization. *EBNA2* induced *IMPDH2* expression predominantly through *MYC*-dependent mechanisms. *IMPDH2* increased the levels of GTP, rRNA/tRNA, and *GNL3*, leading to immortalization. The data are represented as the mean ± standard deviation (SD). Two-tailed Student’s *t* tests were used to indicate between-group differences. The *P* values are shown in each graph.

Cancerous cells typically have larger and more nucleoli, and enlarged nuclei and cells, compared to normal cells (19–21), although the underlying mechanisms are unclear. Recently, Kofuji et al. (22) found that IMPDH2, which is a critical enzyme that is involved in GTP synthesis, is overexpressed in glioblastomas. This leads to nucleolar hypertrophy, the increased synthesis of pre-rRNA and pre-tRNA, and cell proliferation. Our RNA-seq data (12) have shown that *IMPDH2* expression is increased by 2 dpi, and this is followed by a slight gradual decrease, although the level remains high (Fig. 2A). Based on our RNA-seq data (Fig. 2A) and the results of previous studies (22), we hypothesized that *IMPDH2* may play a crucial role in the nucleolar enlargement and growth transformation of B cells via EBV infection. GTP and ATP levels were significantly increased by EBV infection (Fig. 2B; Fig. S1A), and this is in line with previous results for glioblastoma (22). The synthesis of rRNA and tRNA was markedly upregulated at 2 dpi (Fig. 2C and D). We found that the level of *GNL3* (also known as nucleostemin), which is a nucleolar GTP-binding protein that is stabilized by GTP, was increased by EBV (Fig. 2E). Therefore, the EBV infection of primary B cells caused prompt *IMPDH2* induction, and this led to the increased biosynthesis of GTP and other molecules that are required for cell proliferation within two days.

Next, we analyzed the underlying mechanism of *IMPDH2* induction by EBV infection. *EBNA2* is the key molecule that is expressed immediately after infection (Fig. 2F and H), and it regulates the transcription of viral and other cellular genes. The *MYC* gene has a low expression in primary B cells, whereas *EBNA2* induces *MYC* gene expression by 2 dpi (Fig. 2G and I) (23, 24). A previous study reported similar induction patterns for *IMPDH2*, *EBNA2*, and *MYC* after EBV infection in primary B cells (Fig. S1B–D), although *IMPDH2* induction was not evaluated in detail (10). Because *MYC* reportedly induces *IMPDH2* in melanoma (25), we used a specific inhibitor of *MYC* (10058-F4). The inhibitor reduced the *IMPDH2* level in a dose-dependent manner (Fig. S2A). *EBNA2* gene disruption in the EBV genome decreased the induction of *IMPDH2* transcription (Fig. 2J) and MYC (Fig. 2K). Because the bromodomain (BRD) inhibitor JQ1, which typically inhibits super-enhancer activity, decreased the *IMPDH2* induction caused by EBV infection (Fig. S2B), we assume that the *IMPDH2* gene is induced in a manner that is dependent on a super-enhancer involving both *EBNA2* and *MYC*. Therefore, we checked the accumulation of transcriptional activators/regulators, including EBNA2, EBNA-LP, EBNA3A/C, RelA, RelB, cRel, p50, p52, and MYC, and CTCF at the genomic region spanning the *IMPDH2* locus in LCLs (Fig. S2C) (26–28). The ChIP-seq data show EBNA2, EBNA-LP, and MYC accumulation at the promoter region of the IMPDH2 (Fig. S2C, yellow) as well as active epigenetic marker H2K27 acetylation. Furthermore, a ChIA-PET analysis revealed that the *IMPDH2* promoter region organizes 3D connections with several upstream and downstream enhancers, thereby suggesting that the transcriptional upregulation of the *IMPDH2* gene by EBV is mediated by the super-enhancer.

To further explore the effects of *EBNA2* and *MYC* on *IMPDH2* induction, we constructed a correlation chart using the RNA-seq data. The levels of *IMPDH2* and *MYC* were strongly correlated (Fig. 2L), suggesting that *MYC* is important for *IMPDH2* induction (Fig. 2L; Table 1). However, the *IMPDH2* level was poorly correlated with *EBNA2* (Fig. S2D; Table 1). These results are explained by the high level of *IMPDH2* gene expression in the restricted latency patterns of EBV-negative cancerous cell lines or EBV-positive cell lines, such as NK/T cell lines (Fig. S2D; Table 1). In addition, the exogenous expression of *EBNA2* in *EBNA2*-negative P3HR1 cells failed to induce *IMPDH2* gene expression, although the *MYC* level was intrinsically high, even without *EBNA2* (Fig. S2E–G). Taken together, our results suggest that *MYC* is predominantly responsible for *IMPDH2* gene induction and that *EBNA2* plays an indirect or supplemental role, at least in cases of EBV primary infections and lymphoma cell lines. Fig. 2M presents our hypothesis regarding the mechanism underlying *IMPDH2* induction, nucleolar hypertrophy, and immortalization of B cells by an EBV infection.

**TABLE 1:** LogFPKM values from our RNA-seq data

Sample	Cell line	IMPDH2	MYC	EBNA2
Burkitt lymphoma cell lines	Akata-EBV(+)	2.48772788	2.012525095	0.351280179
	Daudi	2.52555011	2.298001111	ND^*a*^
	Mutu I	2.350056033	2.051194985	0.175097035
	Namalwa	2.159043559	2.128803001	1.228780072
	P32ISH	2.174908369	2.331324025	−1.130361173
	P3HR1	2.511808522	2.825616932	ND
	Raji	2.464719004	2.017434042	1.239296976
NK/T cell lines	KAI3	2.248720983	1.606908863	−1.903503632
	MT2-9-7	1.803749278	1.4442978	0.135501565
	MT2-9-9	2.257736157	1.83320004	0.551646362
	SNK10	1.722637213	1.652300504	ND
	SNK6	2.266577395	1.939534228	−1.029315212
	SNT13	1.782174392	1.413618242	−0.157068903
	SNT15	2.36343898	1.650102472	−1.842346831
	SNT16	2.38585284	1.726060997	ND
	SNT8	2.376613451	1.976951246	ND
EBV(+) primary B cells	LCL	2.317624643	1.974538404	2.208817439
	Pre-latent	2.340210048	2.468748941	1.603885614
Primary lymphocytes	Primary B	1.717053567	1.175931989	ND
	Primary NK	1.139781517	0.353683545	ND
	Primary T	1.683865299	1.458064958	ND

a ND, not detected.

**FIG 3:**
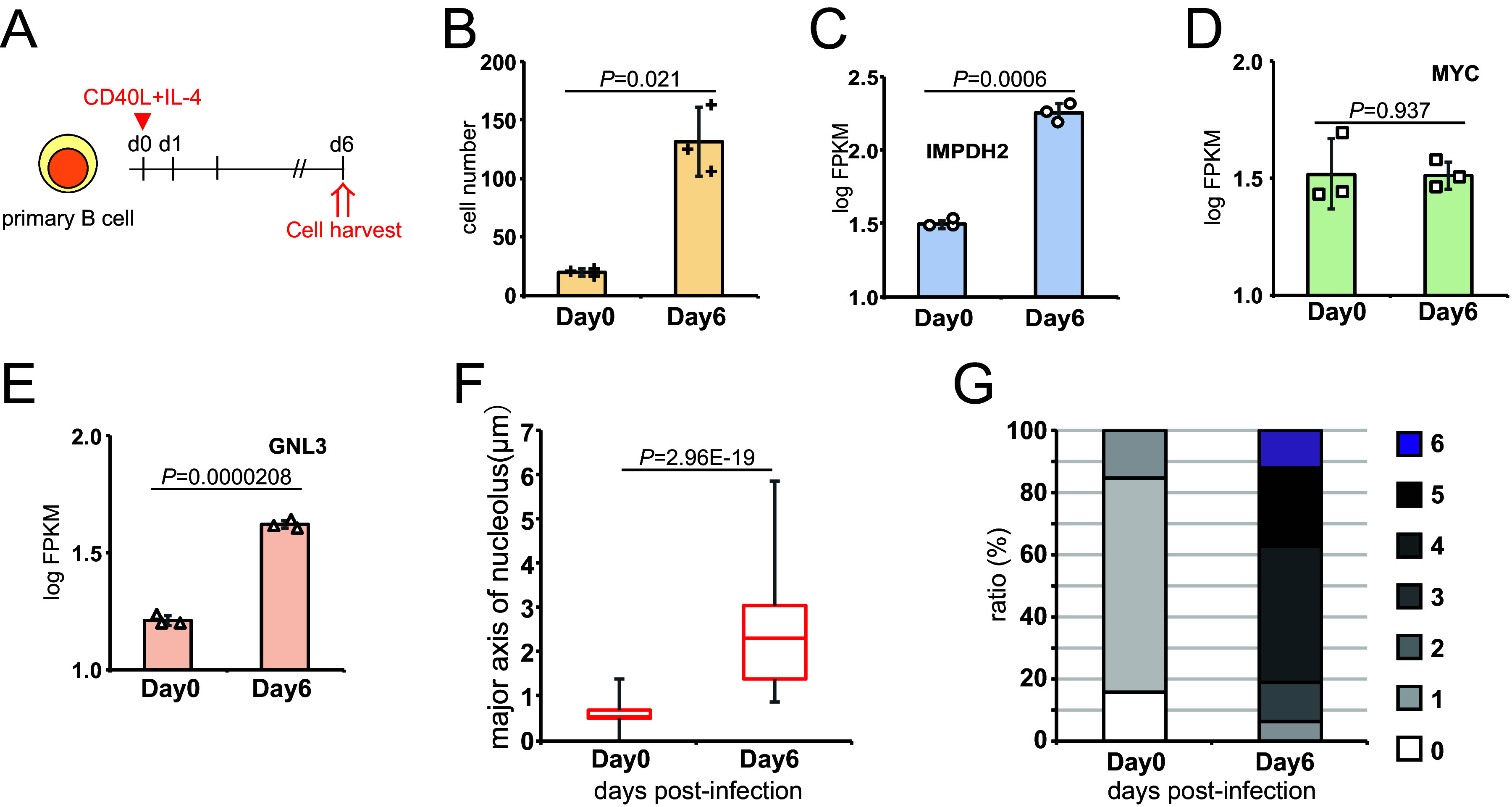
CD40L and IL-4 stimulation of primary B cells induced *IMPDH2* and nucleolar hypertrophy independently of MYC. (A) Primary B cells were treated with CD40L and IL-4 and were harvested six days later. (B) The cell numbers were calculated at days 0 and 6. (C–E) RNA-seq results of *IMPDH2* (C), *MYC* (D), and *GNL3* (E) genes in cells stimulated with CD40L and IL-4 for 0 and 6 days. (F) The major axes of nucleoli were measured using the Zen software package. (G) Number of nucleoli per cell. The data are represented as the mean ± SD. Two-tailed Student’s *t* tests were used to indicate between-group differences. The *P* values are shown in each graph.

To clarify whether *IMPDH2* induction is involved in the B cell proliferation in the absence of EBV, we used CD40L and IL-4 to transiently increase the proliferation of primary B cells (Fig. 3A). The cell number was successfully increased by 6.7-fold at 6 dpi (Fig. 3B). The levels of *IMPDH2* and *GNL3* were significantly increased by CD40L and IL-4, but the *MYC* expression remained unchanged (Fig. 3C–E). Increased nucleolar sizes and numbers were also observed (Fig. 3F and G), suggesting that IMPDH2 is the key molecule that is involved in the cell proliferation and nucleolar hypertrophy of primary B cells with or without an EBV infection, whereas *MYC* is not required to induce *IMPDH2* in the case of activation by CD40L and IL-4. Therefore, *IMPDH2* induction may be regulated by several pathways. An EBV infection stimulates the *EBNA2*-*MYC* axis to induce *IMPDH2*, whereas CD40L and IL-4 activate alternative pathways besides *MYC*.

### Effect of IMPDH inhibition.

**FIG 4:**
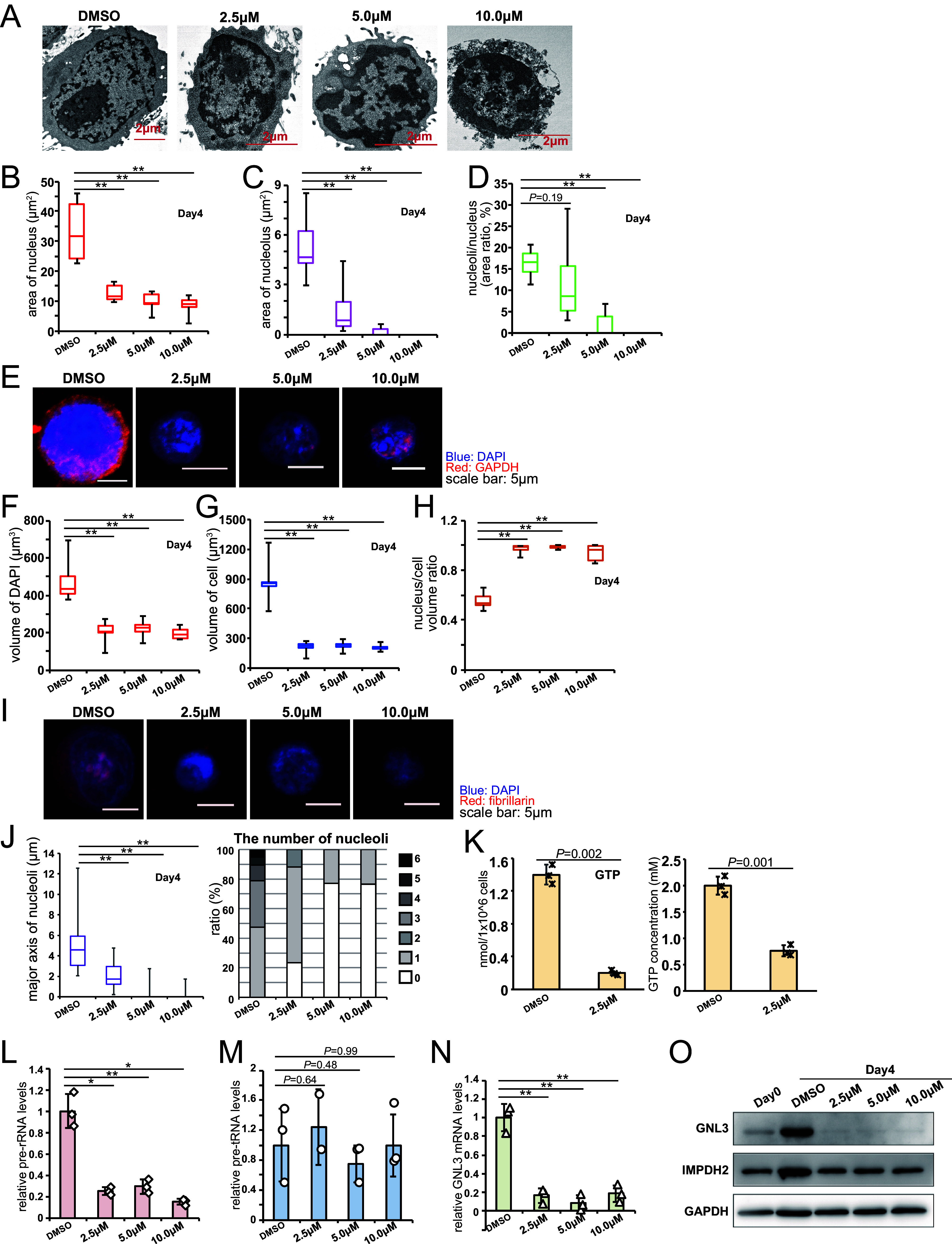
The IMPDH inhibitor MPA reduced GTP synthesis and nucleolar hypertrophy, thereby preventing B cell immortalization by EBV. (A–O) Primary B cells were infected with EBV at a MOI of 3 and were harvested at the indicated time points. (A–D) Morphological changes were analyzed via TEM. (A) Representative images of MPA-treated cells infected with EBV. (B–D) Areas of nucleus (panel B) and nucleolus (panel C) were quantified using the ImageJ software package (*n* = 10 to 15). The nucleoli/nucleus area ratio is shown in (panel D). (E–J) Cell morphology was analyzed using immunofluorescence assays. Serial stacked sections were captured using an LSM710 confocal laser scanning microscope. The images were reconstituted for three-dimensional visualization using the Imaris software package for the volume quantification (*n* = 10 to 15). (E and I) Typical images of EBV-infected cells. (F–H) Volumes of nucleus (panel F, DAPI) and cell (panel G, DAPI + GAPDH). The nucleus/cell volume ratio is shown in (panel H). (J) The major axes of nucleoli (fibrillarin) and the number of nucleoli per cell were calculated using the Zen software package. (K) GTP levels in infected primary B cells were measured using high performance liquid chromatography (HPLC) at 2 dpi. (L–N) qRT-PCR analysis of infected primary B cells at 2 dpi. (O) Western blotting of proteins in EBV-infected primary B cells at 2 dpi. The data are represented as the mean ± SD. Two-tailed Student’s *t* tests were used to indicate between-group differences. *, *P* < 0.05; **, *P* < 0.01.

To provide further evidence that *IMPDH2* is involved in the EBV-driven nucleolar enlargement of primary B cells, the effects of a specific inhibitor of the enzyme mycophenolic acid (MPA) were examined (Fig. 4). A TEM analysis (Fig. 4A) and the quantification of the area (Fig. 4B–D) revealed that MPA inhibited the enlargement of the nuclei and nucleoli of primary B cells after infection in a dose-dependent manner. Fig. 4E presents representative 3D images of an immunofluorescence assay of infected B cells that were treated with MPA at 4 dpi (data from 2 dpi are presented in Fig. S3A–F). The DAPI-positive volume (nuclear volume) was increased to 400 μm^3^, which corresponded to the previously analyzed nuclear size, by infection at 4 dpi (Fig. 4F, DMSO) (Fig. 1F). The sizes of the MPA-treated cell nuclei (almost 200 μm^3^) (Fig. 4F) were similar to the nuclear sizes on day 0 (Fig. 1F). Similarly, the cell volumes were not increased (Fig. 4G) and the nucleus/cell volume ratios were marginally increased (Fig. 4H) with MPA treatment. MPA treatment decreased the nucleolar sizes and numbers in a dose-dependent manner (Fig. 4I and J) that was associated with a lower GTP level (Fig. 4K) as well as decreased expressions of pre-rRNA (Fig. 4L) and GNL3 (Fig. 4N), although tRNA synthesis was not affected by MPA due to unknown reasons (Fig. 4M). Western blotting showed that the protein levels of GNL3 and IMPDH2 were increased by EBV infection and decreased by MPA treatment (Fig. 4O).

**FIG 5:**
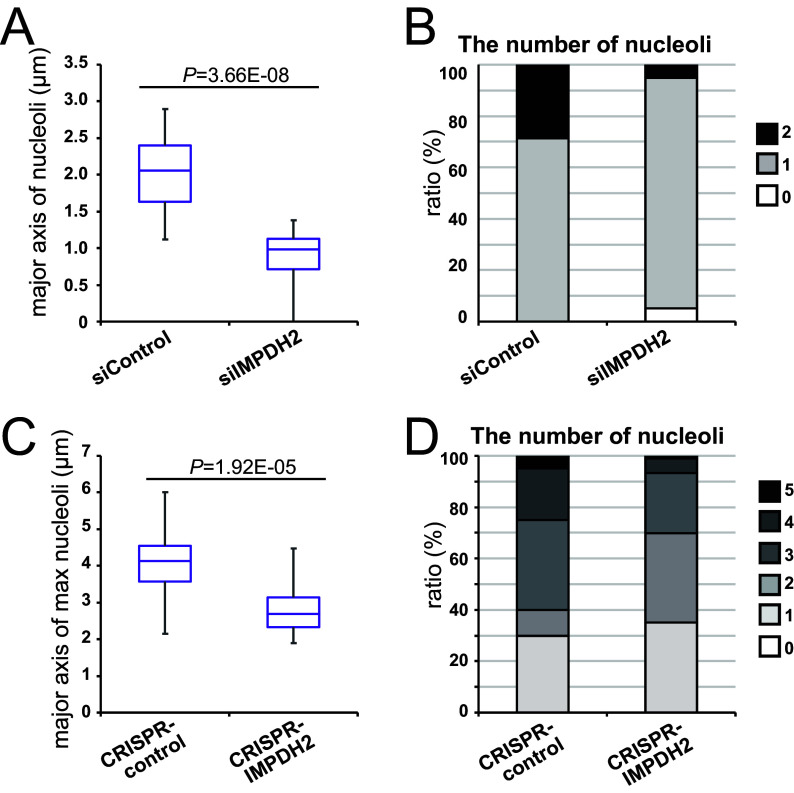
Knockdown of IMPDH2 reduced hypertrophy and the numbers of nucleoli in EBV-infected primary B and B95-8 cells. (A and B) Primary B cells were transfected with control siRNA (siControl) or siRNA for IMPDH2 (siIMPDH2), infected with EBV at a MOI of 3, and harvested at 2 dpi for nucleolin and IMPDH2 staining via immunofluorescence. Serially stacked sections were captured using an LSM710 confocal laser scanning microscope. The images were reconstituted for three-dimensional visualization using the Imaris software package. The major axes of nucleoli (A) and numbers of nucleoli per cell (B) were calculated using the Zen software package (*n* = 15 to 26). The fluorescence intensity of IMPDH2 was measured using the Zen software package, and the cutoff value was determined according to a receiver operating characteristic (ROC) curve analysis. (A) The nucleolar major axes of the siControl-treated cells that expressed IMPDH2 at levels that were above the cutoff and those of the siIMPDH2-treated cells that expressed IMPDH2 at levels that were below the cutoff (siIMPDH2) are compared. (B) Likewise, the nucleolar numbers per cell of the siControl-treated and siIMPDH2-treated cells are shown. (C, D) Similar experiments were carried out as in panels A and B, except that we here transfected CRISPR/Cas9 vectors to Tet-Z/B95-8 cells and harvested at 5 days after the transfection. The major axes (panel C) and the numbers of the largest nucleoli in each cell (panel D) are plotted. The *P* values of two-tailed Student’s *t* tests are shown. Here, we set thresholds of IMPDH2 levels because the transfection of the siRNA and CRISPR/Cas9 vectors were inefficient. The results of the total cell counts are also presented (Fig. S4).

To further evaluate the effect of *IMPDH2* on nucleolar hypertrophy at the cell culture level, we transfected primary B cells with a control siRNA and a siRNA for *IMPDH2*, and this was followed by EBV infection. After two days, the sizes of nucleoli in the IMPDH2-abated cells appeared significantly smaller (Fig. 5A; Fig. S4A, C, and D), and the number of nucleoli per cell was less (Fig. 5B; Fig. S4B) than that observed in the control. The transduction of the CRISPR/Cas9 vector for the *IMPDH2* gene also caused shorter major axes (Fig. 5C; Fig. S4E) and smaller numbers of nucleoli (Fig. 5D; Fig. S4F).

**FIG 6:**
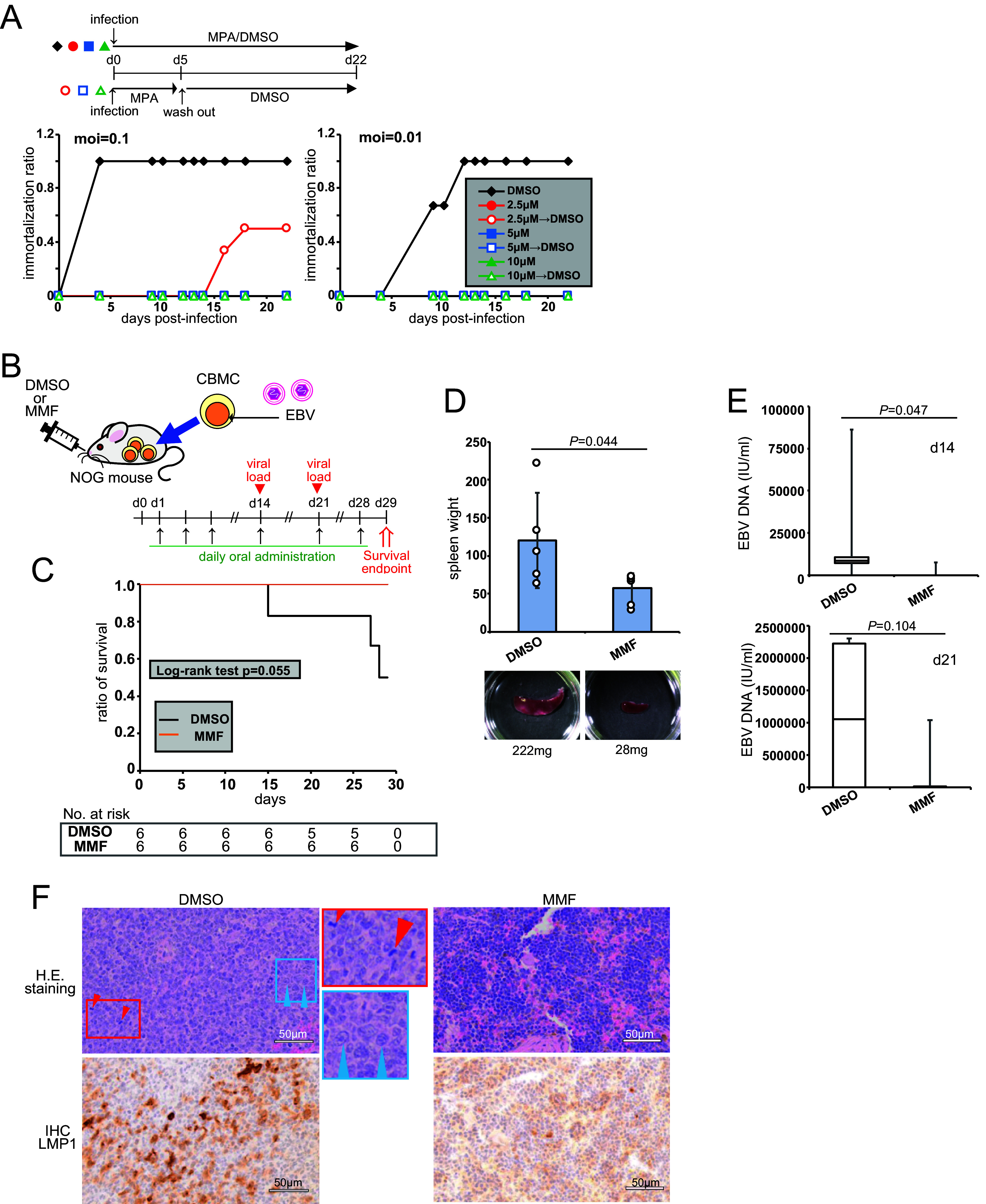
An IMPDH inhibitor decreased tumor formation and pathogenicity in a mouse xenograft model. (A) PBMCs were infected with EBV at a MOI of 0.1 or 0.01, and the number of wells with a clump formation was counted. MPA was added at the indicated concentration for 22 days or was added for the first 5 days and followed by the addition of DMSO. (B–F) Human CBMCs were infected with EBV and injected into NOG mice (*n* = 5 to 6). (B) Schematic diagram of the experiment. The mice were orally administered DMSO or MMF daily for 28 days. (C) Survival of mice. Statistical significance was estimated via the log-rank test. (D) At day 29, the mice were sacrificed, and their spleens were weighed (upper panel). The data are represented as the mean ± SD. (E) The EBV DNA load in the peripheral blood. (F) H&E and LMP1 staining of the pancreases of the mice. Areas surrounded by red and blue rectangles are enlarged to show mitotic cells (red arrowheads) and large, atypical lymphocytes with nuclear enlargement and prominent multiple nucleoli (blue arrowheads), respectively. Two-tailed Student’s *t* tests were used to indicate between-group differences. The *P* values are shown in each graph.

Next, we examined the effects of an IMPDH inhibitor on B cell growth transformation by EBV. Peripheral blood mononuclear cells (PBMCs) were infected with EBV at a MOI of 0.1, 0.01, or 0.001 in the presence or absence (DMSO) of MPA (Fig. 6A; Fig. S5). All wells without MPA treatment showed cell clumping, suggesting growth transformation, by days 5 and 12, at a MOI of 0.1 or 0.01, respectively. Continuous MPA treatment, even at the lowest concentration (2.5 μM), blocked clump formation. Similar results were obtained at a MOI of 0.001 (Fig. S5). To test the early effects of MPA after infection, we treated cells with MPA for 5 days. Treatment with 2.5 μM MPA for 5 days that was followed by the removal of the inhibitor caused a slight increase in clump formation at a MOI of 0.1 (Fig. 6A). Therefore, MPA treatment efficiently inhibited *in vitro* EBV-driven immortalization.

We intraperitoneally injected EBV-infected human cord blood mononuclear cells (CBMCs) into NOD/Shi-scid-IL2Rγ^null^ (NOG) mice. The mice were administered daily with mycophenolate mofetil (MMF), a prodrug of MPA, or DMSO orally for four weeks (Fig. 6B). The longevity and body weights of the NOG mice were observed (Fig. 6C; Fig. S6A). At the time of sacrifice on day 29, measurements of spleen sizes suggested that MMF inhibited splenomegaly (Fig. 6D). Peripheral blood was collected at days 14 and 21 to analyze EBV DNA. MMF reduced the viral load from the peripheral blood at day 14 (Fig. 6E). The hematoxylin and eosin (H&E) staining of spleen sections (Fig. 6F, upper panels) showed that MMF treatment was associated with reduced mitosis (red arrowheads) and large, atypical lymphocytes with nuclear enlargement and prominent multiple nucleoli (blue arrowheads). Immunohistochemistry showed that the spleens of the DMSO-treated mice were strongly stained with LMP1, whereas MMF treatment suppressed the staining (Fig. 6F, lower panels). The administration of MMF for the first 14 days after injection also improved the survival of the mice (Fig. S6B and C). MMF is a prodrug of MPA and is clinically used for the anti-rejection of posttransplant patients as well as the mitigation of autoimmune disorders, including systemic lupus erythematosus (SLE) (29). MMF treatment significantly improved mouse survival (Fig. 6C; Fig. S6C), indicating that IMPDH inhibitors decreased lymphomagenesis *in vivo*.

## DISCUSSION

Because primary B cells are in the resting (G_0_) state, EBV alters the gene expression machinery to increase the efficiency of growth transformation (30). This is accomplished partly through epigenetic changes, including CpG demethylation and histone acetylation, which result in the drastic reduction of nuclear heterochromatin (31–33). EBV infection triggers the formation of superenhancers that induce specific host genes, such as *MYC*, in which viral gene products, such as EBNA2 and EBNA3, are involved (13, 27). Furthermore, previous studies have used gene editing to identify the factors that are essential for EBV growth transformation, such as IRF2, IRF4, BATF, SYK, CFLAR, RBPJ, CCND2, CDK4, CDK6, CD19, CD81, and RelA (34). In addition, studies have found that EBV subverts host mevalonate and fatty acid pathways (35) as well as induces cytidine metabolism through the *CTPS1* and *CTPS2* genes (36).

In the present study, we found that EBV infections increased the numbers and sizes of nucleoli (Fig. 1C, K, and L). Nucleoli are nuclear bodies that are not surrounded by a membrane and are located at chromosomal regions called nucleolus organizer regions (NORs), which include rRNA gene repeats that encode rRNA sequences (37, 38). They are the site of rRNA transcription, rRNA storage, and ribosome assembly. Increases in their sizes and numbers are pathological features of tumor cells, including EBV-associated B cell lymphomas. However, the underlying mechanisms have remained unclear for more than 100 years. In 2019, the rate-limiting enzyme for GTP synthesis, namely, *IMPDH2*, was identified, and it appears to be the bottleneck in nucleolar enlargement and increased biosynthesis (22, 39, 40). In a glioblastoma model, an increased GTP level due to *IMPDH2* induction in cancer cells caused the transcriptional activation of ribosomal RNAs, thereby increasing ribosomal biogenesis, upregulating cellular genes, and promoting cellular proliferation.

A RNA-seq analysis showed that *IMPDH2*, which was expressed at low levels in primary B cells, was rapidly induced by EBV infection (Fig. 2A). Indeed, transcriptome data from previous studies have shown that *IMPDH2* is induced by the EBV infection of primary B cells by 2 dpi (11, 14). In addition, *IMPDH2* induction by EBV infection is significantly alleviated by the deletion of the viral *EBNA2* gene (Fig. 2J), indicating that *EBNA2* is required for *IMPDH2* expression immediately after EBV infection. These results are in line with those of a previous study that used *EBNA2* that was C-terminally fused to an estrogen receptor; *EBNA2* ablation by 4-hydrohytamoxifen depletion decreased *IMPDH2* gene expression in LCLs (24). *EBNA2* induces the *MYC* gene (41), which is a transcriptional activator of *IMPDH2* (25, 42). The expression levels of *IMPDH2* in primary lymphocytes and lymphomas are strongly correlated with those of *MYC* but not *EBNA2* (Fig. 2L; Fig. S2D). In addition, *EBNA2* overexpression did not increase the expression levels of *IMPDH2* and *MYC* in P3HR1 cells (Fig. S2E–G). Treatment with the MYC inhibitor attenuated *IMPDH2* induction in primary B cells (Fig. S2A), suggesting that *MYC*, but not *EBNA2*, is the major transcriptional activator of *IMPDH2*. However, interestingly, the transient mitogenic activation of primary B cells by CD40L and IL-4 was associated with the increased expression of *IMPDH2* but not *MYC* (Fig. 3). Therefore, CD40L and IL-4 increase *IMPDH2* expression independently of *MYC*. There may be multiple pathways for *IMPDH2* gene induction because of its central role in cellular proliferation. Interestingly, although the *MYC* gene was not increased by CD40L or IL-4 treatment, the *MYCL* expression was significantly induced (data not shown). Therefore, *MYCL* may play a significant role in *IMPDH2* expression.

The EBV-induced *IMPDH2* gene was associated with an increased GTP level (Fig. 2B), which correlated with the biosynthesis of rRNA, tRNA, and the nucleolar protein GNL3 as well as nucleolar hypertrophy by 2 dpi. The sizes of the nuclei and cells were increased by EBV infection slightly after (4 dpi) the increase in nucleolar size (Fig. 1). Several studies have reported efficient cellular proliferation after EBV infection at 4 to 8 dpi (43, 44). Therefore, we speculate that rapid cell division requires abundant rRNA, tRNA, nucleolar proteins, and ribosomes that support the efficient *de novo* synthesis of viral and cellular proteins.

Furthermore, the administration of a specific IMPDH inhibitor, namely, MPA, blocked the EBV-induced biosynthesis of GTP, rRNA, tRNA, and *GNL3* as well as the enlargement of the nucleolus, nucleus, and cell (Fig. 4). MPA also suppressed *in vitro* growth transformation by EBV infection (Fig. 6A), which is in line with previous data that show the MPA treatment of LCL causing cell growth inhibition and cell death (45–47). MMF treatment in a mouse xenograft model delayed tumor formation and prolonged survival (Fig. 6B–D). This finding is important because MMF is approved for use as an immunosuppressant (29). Our results provide basic evidence that MMF use may prevent PTLD after transplants. In fact, previous studies have found that the use of MMF in transplant patients reduces the risk and incidence of PTLD (48–55) or, at least, does not increase the risk for PTLD (56–59). However, MMF increases the incidence of PTLD involving the central nerve system (CNS) (60, 61). The underlying reasons for the increased risk for CNS PTLD with MMF use are unclear. However, the transport of MPA through the blood-brain barrier is inefficient because >95% of the MPA, which is the active metabolite of MMF, forms a complex with serum albumin (62, 63).

Increased *IMPDH2* expression is present in various human cancers and may be related to a poor prognosis. For instance, four cohorts of glioma patients showed that higher *IMPDH2* expression was positively correlated with the risk of malignancy, a poor prognosis, and even chemoresistance (22, 64). Higher *IMPDH2* expression in osteosarcoma is associated with a poor prognosis and even resistance to chemotherapy and radiotherapy (47, 65, 66). Similar results have been obtained for several other cancers (67–75). Clinical trials are already underway to examine the anti-cancer effects of MPA and MMF (76). For instance, multiple myeloma cells express higher levels of *IMPDH2*, and MMF use results in a positive clinical response (77). Therefore, IMPDH inhibitors may be used for several cancers in addition to PTLD.

Viruses may use *IMPDH2* to increase biogenesis in infected cells. In fact, MPA and MMF also have potential antiviral effects against several viruses, such as influenza virus (78), the hepatitis B (79) and C (80) viruses, human immunodeficiency virus (81), rotavirus (82), norovirus (83), coxsackie virus (84), dengue virus (85), Chikungunya virus (86), and SARS-CoV-2 (87). Although MPA and MMF have immunosuppressive effects, *IMPDH2* is an attractive target for antiviral drugs.

Taken together, our results suggest that EBV increases *IMPDH2* expression and thereby increases the GTP level, leading to nucleolar hypertrophy and the increased biogenesis of ribosomes. This exploitation of host biogenesis by EBV is required for B cell growth transformation. Our study provides basic, mechanistic evidence that the use of MMF in patients with transplants or SLE prevents PTLD. MMF may also be used for other cancers and viral infections, but further studies are required to confirm its usefulness.

## MATERIALS AND METHODS

### Cells, virus, and reagents.

Primary B cells from PBMCs of healthy donors (LONZA, no. 4W-601) were cultured in RPMI 1640 medium (Sigma-Aldrich, St. Louis, MO, USA) supplemented with 10% fetal bovine serum. EBV was produced using AGS/EGFP-EBV cells (88) and titrated according to GFP positivity after the infection of EBV-negative Akata cells. EBV infection was performed by inoculating EBV with primary B cells and changing half of the medium every few days. The anti-nucleostemin (Cell Signaling Technology, Danvers, MA, USA; no. 9495), anti-IMPDH2 (Abcam, Cambridge, MA, USA; no. ab131158), anti-GAPDH (Ambion Inc., Austin, TX, USA; AM4300), anti-GNL3 (nucleolin) (MBL, Watertown, MA, USA; M019-3), and anti-fibrillarin (Abcam; no. 4566) antibodies were purchased. MPA and MMF were purchased from FUJIFILM Wako (Osaka, Japan).

### TEM.

The primary B cells that were infected with wild-type EBV were harvested at the indicated time points. The cells were washed with phosphate-buffered saline (PBS) and fixed in half-strength Karnovky’s fixative (2% paraformaldehyde and 2.5% glutaraldehyde in 0.1 M sodium phosphate [PB]) for 1 h at 4°C. The specimens were washed with PBS twice at room temperature (RT) for 5 min and were postfixed with 2% osmium tetroxide. Following postfixation, the specimens were washed with MQ twice at RT for 5 min, dehydrated in a graded ethanol series (50%, 70%, 80%, 90%, 100%, 100%, and 100% for 5 min each at RT), suspended in propylene oxide (QY-1) for 30 min at RT, and embedded in Epon 812 (Taab Laboratories, Aldermaston, UK) at 60°C. The embedded cells were sectioned using an ultracut microtome. The ultrathin sections were stained with 2% uranyl acetate solution and Sato lead solution. TEM was performed using a JEM-1400 Flash (JEOL, Tokyo, Japan) at an accelerating voltage of 80 kV.

### Immunofluorescence analysis.

The primary B cells that were infected with wild-type EBV were harvested at the indicated time points. The cells were fixed with 70% ethanol overnight at −20°C, postfixed with 4% paraformaldehyde at RT for 15 min, washed with wash buffer (0.5% bovine serum albumin in PBS-T) three times, and blocked with 5% bovine serum albumin in PBS-T for 30 min. Then, the cells were stained with the primary antibody overnight at 4°C, and this was followed by staining with the secondary antibody for 1 h at 37°C. The primary and secondary antibodies were diluted with wash buffer. The stained cells were mounted on ProLong Diamond with DAPI (ThermoFisher Scientific, Waltham, MA, USA) for nuclear staining.

### Confocal microscopy.

Confocal microscopy was performed on a Zeiss LSM-710 system (Carl Zeiss, Jena, Germany). Images were photographed using a Plan-Apochromat 63×/1.4 lens objective. For the 3D reconstruction, Z-stacks were performed at a Z-step of 0.35 μm.

### Microscopic data analysis.

To analyze TEM images, the areas of the nuclei and nucleoli were measured using ImageJ software. The nuclear and cellular volumes were measured using Imaris software. Serial sections were observed using a confocal laser scanning microscope (Zeiss LSM-710) and were reconstructed to a 3D isosurface at a fixed threshold. The long axis of the nucleoli was measured using serial images of the sections and the Zen software package.

### RNA-seq.

Briefly, RNA from primary B cells infected with EBV were collected at the indicated time points, and this was followed by polyA+ RNA isolation and library preparation using a NEBNext Poly(A) mRNA Magnetic Isolation Module and a NEBNext Ultra II Directional RNA Library Prep Kit for Illumina (both from New England Biolab), according to the manufacturer’s instructions. The sequencing was carried out by using a HiSeq X next-generation sequencer or a NextSeq 2000 using P2 Reagents (100 cycles) (Illumina).

### Quantitative RT-PCR and Western blotting.

For the qRT-PCR, total RNA was isolated from cells using TriPure Isolation Reagent (Roche, Rotkreuz, Switzerland). The qRT-PCRs were performed using a One-Step TB Green PrimeScript PLUS RT-PCR Kit (TaKaRa, Shiga, Japan) and a Step One Plus real-time PCR system (ThermoFisher Scientific). The primer sequences used in this study were as follows: *IMPDH2*, 5′-CTGTTTCTTGGAAGAGATAATG-3′ and 5′-CTTGCTGCGCTGCAGAATTTC-3′; *GAPDH*, 5′-GAGTCAACGGATTTGGTCGT-3′ and 5′-TTGATTTTGGAGGGATCTCG-3′; 18S rRNA, 5′-CGCCGCTAGAGGTGAAATTCT-3′ and 5′-CGAACCTCCGACTTTCGTTCT-3′; pre-rRNA, 5′-TGTCAGGCGTTCTCGTCTC-3′ and 5′-AGCACGACGTCACCACATC-3′; pre-tRNA, 5′-CACCCTGATAGAGCCATCAC-3′ and 5′-CTCTGCATGTACTGCTGTATAAGTACC-3′; *GNL3*, 5′-AAAGCCATTCGGGTTGGAGT-3′ and 5′-CCACAGCAGTTTGGCAGCAC-3′; *EBNA2*, 5′- TTAGAGAGTGGCTGCTACGCATT-3′ and 5′- TCACAAATCACCTGGCTAAG −3′; and *MYC*, 5′-AGAGTTTCATCTGCGACCCG-3′ and 5′-AAGCCGCTCCACATACAGTC-3′. The Western blotting was performed as described previously (12).

### High-performance liquid chromatography (HPLC) for cellular ATP and GTP.

The cell pellets were rinsed with ice-cold PBS, and the supernatant was removed via centrifugation at 16,000 × *g*. Then, 1 mL 80% methanol was added, and the solution was mixed. The samples were centrifuged at 16,000 × *g* for 10 min, and the supernatant was transferred to a new test tube. The samples were evaporated and resuspended with 100 μL mobile phase A buffer (20 mM phosphate buffer, pH 8.5) containing 10 mM tetrabutylammonium (Tokyo Chemical Industry Co., Ltd., Tokyo, Japan). After centrifugation at 16,000 × *g* for 10 min, the supernatant (70 μL) was transferred to a new test tube. Then, 10 μL of supernatant were injected into an HPLC system (Alliance e2695; Waters, Milford, MA, USA) with an XBridge BEH C18 column (3.5 μm; 4.6 × 50 mm; Waters). The mobile phase B buffer consisted of CH_3_CN. The flow rate was set to 1.0 mL/min. The linear gradient program used was as follows: 5.5 to 6% for 0 to 15 min, 6 to 6.5% for 15 to 20 min, 10% for 20 to 25 min, and 5.5% for 25 to 45 min. The UV detection was set at a wavelength of 254 nm using a 2998 PDA Detector (Waters). Data were collected and processed using the Empower 3 software package for the HPLC system (Waters). The peaks were identified by comparing the retention times and the similarity between the chromatographic peak spectrum with the reference standards of ATP and GTP (Wako). To calculate the concentrations, the measured values were divided by the cell volume.

### *EBNA2* overexpression via electroporation.

*EBNA2* transfection was performed using a Neon transfection system (Invitrogen, Carlsbad, CA, USA). Then, 2.5 μg pSG5-*EBNA2*, *EBNA2* expression vector, or pSG5 were transfected into 1 × 10^6^ P3HR1 cells via electroporation (1,400 V, 20 ms, 2 N). The cells were seeded on a 6-well plate and were cultured with 2 mL RPMI for 72 h.

### CD40L and IL-4 stimulation.

B Cell Expansion Kits (130–106–196; Miltenyi Biotec GmbH, Bergisch Gladbach, Germany) and defibrinated human AB serum (ACC-HAB-7102MH; Access Biologicals, Vista, CA, USA) were used to stimulate primary B cells from PBMCs of healthy donors (LONZA, no. 4W-601), according to the manufacturer’s instructions.

### Ablation of IMPDH2.

The siRNA for IMPDH2 or its control, purchased from Santa Cruz Biotechnology (Dallas, TX, USA), were transfected into 1 × 10^6^ primary B cells via electroporation (1,400 V, 20 ms, 2 N). The cells were infected with EBV at a MOI of 3 and were harvested at 2 dpi. The IMPDH2 CRISPR/Cas9 knockout (KO) plasmid and control CRISPR/Cas9 plasmid were purchased from Vector Builder, and 2.5 μg of each plasmid was transfected into Tet-Z/B95-8 cells utilizing lipofectamine 3000. After 5 days, the cells were harvested. Those cells were subjected to fixation and immunofluorescence assays, as mentioned above.

### B cell growth transformation assay.

An immortalization assay was performed as described previously (89). In brief, PBMCs from healthy donors (LONZA no. CC-2702) were seeded on 96-well plates with cyclosporine in the presence of DMSO or MPA. EBV that was obtained as previously mentioned was subjected to 10-fold serial dilutions and was mixed with PBMCs in the plates. Clump formation was examined under a microscope at the indicated time points.

### Mouse xenograft model.

The experiments on EBV-associated lymphoproliferative disorder, using a mouse model, were performed as described previously (90). Immunodeficient NOG mice (NOD/Shi-scid, IL-2RgKO) and human CBMCs were obtained from the Central Institute for Experimental Animals and RIKEN, respectively. Human CBMCs were inoculated with EBV *in vitro* and were intraperitoneally injected into NOG mice. DMSO or MMF (120 mg/kg) was administered orally. Viral DNA in the mouse blood was quantified, and a histological analysis was performed as previously described (91).

### Data availability.

The RNA-seq data presented in Fig. 2 were obtained from a previous study (12) (DDBJ accession no.: DRA011328). The RNA-seq data for Fig. 2L, Fig. S2D (DRA015073), and Fig. S3 (DRA015092) will be available from DDBJ when the manuscript is accepted for publication. The ChIP-seq and ChIA-PET data were obtained from the publicly available EBV regulome resource tracks (26–28).
