# Supplementary material for: Growth Transformation of B Cells by Epstein-Barr Virus Requires IMPDH2 Induction and Nucleolar Hypertrophy
Source: Microbiol Spectr. 2023 Jul 6;11(4):e00440-23. doi: 10.1128/spectrum.00440-23 (PMC10433962; doi:10.1128/spectrum.00440-23)

**Growth Transformation of B cells by Epstein-Barr Virus Requires *IMPDH2* Induction and Nucleolar Hypertrophy**

Atsuko Sugimoto^1,2^, Takahiro Watanabe^3^, Kazuhiro Matsuoka^2^, Yusuke Okuno^4^, Yusuke Yanagi^3^, Yohei Narita^5^, Seiyo Mabuchi^3,6^, Hiroyuki Nobusue^7^, Eiji Sugihara^7^, Masaya Hirayama^8,9^, Tomihiko Ide^1,10^, Takanori Onouchi^10^, Yoshitaka Sato^3^, Teru Kanda^11^, Hideyuki Saya^7^, Yasumasa Iwatani^2^, Hiroshi Kimura^3^, Takayuki Murata^1,3^

**Supplemental Figure legends**

**Fig. S1**: (a) ATP level in primary B cells infected with EBV for 0 and 2 days. The data are represented as the mean ± SD. (b–d) RNA-seq data from a previous study. LogFPKM values of *IMPDH2* (b), *EBNA2* (c), and *MYC* (d) genes were obtained from (10). The two-tailed Student’s t test was used to indicate between-group differences. The P values are shown in each graph.

**Fig. S2**: Effects of MYC inhibitor 10058-F4 (a) and BRD inhibitor JQ1 (b). LCLs were treated with the inhibitors at the indicated concentrations and harvested for qRT-PCR. (cです) Publicly available ChIP-seq and ChIA-PET data of LCLs at the *IMPDH2* loci were obtained and presented. Yellow box indicates the *IMPDH2* gene, and other boxes indicate possible enhancer regions. (d) Correlation between the expressions of *IMPDH2* and *EBNA2* were plotted using peason correlation analysis. The LogFPKM values are presented in Table 1. (e–g) P3HR1 cells were transfected with the *EBNA2* expression vector. After 72 h, cells were harvested and RNAs were extracted. RNA-seq analyses were performed and LogFPKM values were calculated for *EBNA2* (e), *MYC* (f), and *IMPDH2* (g). The data are represented as the mean ± SD. The two-tailed Student’s t test was used to indicate between-group differences. ns: not significant, *:P<0.05, **:P<0.01

**Fig. S3**: (a–f) Effect of IMPDH inhibitor MPA on EBV-infected primary B cells. Primary B cells were infected with EBV at an MOI of 3 and harvested at day 2. Cell morphology was analyzed via immunofluorescence assays. Serial stacked sections were photographed using an LSM710 confocal laser scanning microscope. The images were reconstituted for 3D visualization using Imaris software for volume quantification (n = 10–15). (a, e) Representative 3D images of EBV-infected cells. Volumes of nucleus (b, DAPI) and cell (c, DAPI+GAPDH). (d) The nucleoli/nucleus area ratio is shown. (e) The major axes of nucleoli (fibrillarin) and the number of nucleoli per cell were calculated using Zen software. (f) ATP level in infected primary B cells treated (or not) with 2.5 μM MPA was analyzed via HPLC. The data are represented as the mean ± SD. The two-tailed Student’s t test was used to indicate between-group differences. ns: not significant, *:P<0.05, **:P<0.01

**Fig. S4**: Knockdown of IMPDH2 reduced nucleolar hypertrophy in EBV-infected primary B and B95-8 cells. (a, b) The same experimental data with Fig. 5a and b, except that data for total cell counts are shown. Primary B cells were transfected with Control siRNA (siControl) or siRNA for IMPDH2 (siIMPDH2), infected with EBV at an MOI of 3, and harvested at 2 dpi for Nucleolin and IMPDH2 staining by immunofluorescence. Serially stacked sections were captured using an LSM710 confocal laser scanning microscope. The images were reconstituted for 3D visualization using Imaris software for volume quantification. The major axes of nucleoli (aです) and number of nucleoli per cell (b) were calculated using Zen software (n = 15–26). (a) Nucleolar major axes of siControl-treated cells and those of siIMPDH2-treated cells are compared. (b) Likewise, Nucleolar number per cell of siControl- and siIMPDH2-treated cells are shown. (c) Typical images of siRNA-transfected cells. (d) The peason correlation chart of the mean of the fluorescence intensity (MOI) of IMPDH2 and the major axis of the largest nucleolus in each primary B cell, transfected with siIMPDH2 and infected with EBV. Cut-off value was determined according to the ROC curve analysis, and the values higher and lower than the cut-off are shown in white and black, respectively. (e, f) Similar experiments were carried out as Fig. S4a and b, except that we here transfected CRISPR/Cas9 vectors to Tet-Z/B95-8 cells and harvested at 5 days after the transfection. Major axes (e) and the number of the largest nucleoli in each cell (f) are plotted. The P values of two-tailed Student’s t test are shown.

**Fig. S5**: Growth transformation by EBV infection at an MOI of 0.001 in the presence or absence of MPA.

**Fig. S6**: (a Raw data of body weights of the mouse experiment (Fig. 5b–f), in which MMF was administered daily until day 28. (b, c) Results of similar experiments except that MMF was administered on days 1–14. (b) Schematic diagram of the experiment (n = 5–6). (c) Survival of mice. Statistical significance was estimated by the log-rank test.

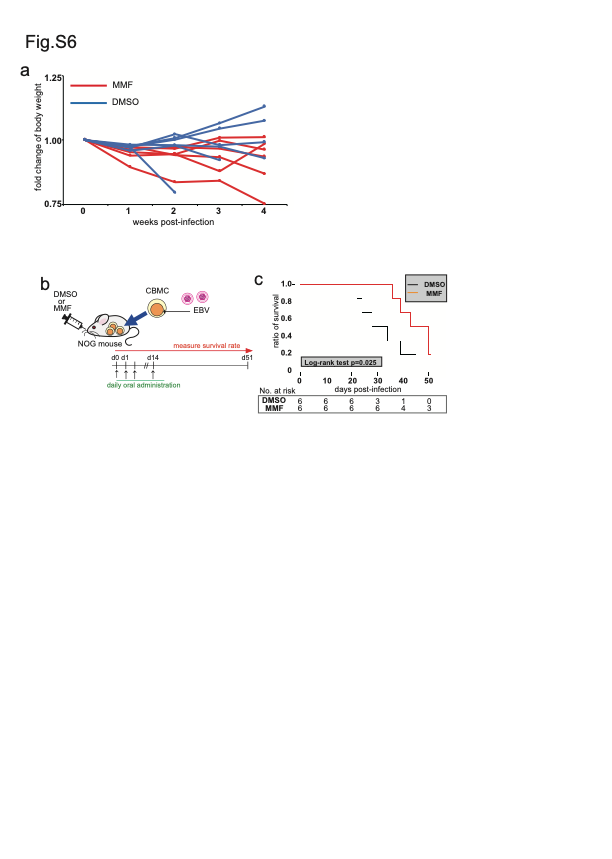

Supplement: Supplemental file 1 — Fig. S1 to S6. Download spectrum.00440-23-s0001.docx, DOCX file, 13.8 MB [file spectrum.00440-23-s0001.docx]
